# Supplementary material for: Integrative genomics of Plasmodium knowlesi reveals parasite-intrinsic regulators of severe human malaria
Source: bioRxiv. 2026 Mar 23:2026.03.22.713451. Preprint. [Version 1] doi: 10.64898/2026.03.22.713451 (PMC13042041; doi:10.64898/2026.03.22.713451)
Supplement: Supplement 1 [file media-1.pdf]

Integrative genomics of *Plasmodium knowlesi* reveals parasite-intrinsic regulators of severe human malaria

Jacob A F Westaway[*, 1, 2], Ernest Diez Benavente[3], Michal Kucharski[4], Sourav Nayak[4], Duong T Q Huy[4], Sarah Auburn[1], Timothy William[5], Giri S Rajahram[5,6], Roberto Amato[8], Kim A Piera[1], Hidayat Trimarsanto[1,12], Kian Soon Hoon[1], Caitlin Bourke[1], Anisah Jantim[7], Abdul Marsudi Manah[8], William Gotulis[8], Robert W Moon[9], Bridget E Barber[1], Chris Drakeley[9], Nicholas M Anstey[1], Matt A Field[†1,2,11,13], Zbynek Bozdech[*†4,] Matthew J Grigg[†1,14]

*Corresponding authors
†Equal contribution

Affiliations

1. Menzies School of Health Research and Charles Darwin University, Northern Territory, Australia
2. Centre for Tropical Bioinformatics and Molecular Biology, James Cook University, Cairns, Queensland, Australia
3. Laboratory of Experimental Cardiology, University Medical Center Utrecht, Utrecht, the Netherlands
4. Nanyang Technological University, Singapore
5. Infectious Diseases Society Sabah-Menzies School of Health Research Clinical Research Unit, Queen Elizabeth Hospital, Kota Kinabalu, Sabah, Malaysia
6. Queen Elizabeth II Hospital-Clinical Research Centre, Ministry of Health Malaysia, Sabah, Malaysia
7. State Public Health Laboratory, Kota Kinabalu, Sabah, Malaysia
8. Sabah State Health Department, Kota Kinabalu, Sabah, Malaysia
9. London School of Hygiene & Tropical Medicine, London, United Kingdom
10. Wellcome Sanger Institute, United Kingdom
11. College of Science and Engineering, James Cook University, Townsville, Queensland, Australia

12. Eijkman Research Center for Molecular Biology, National Research and Innovation Agency, Indonesia
13. Immunogenomics Lab, Garvan Institute of Medical Research, Darlinghurst, NSW, Australia
14. Institute for Health Research, University of Notre Dame Australia, Fremantle, Western Australia

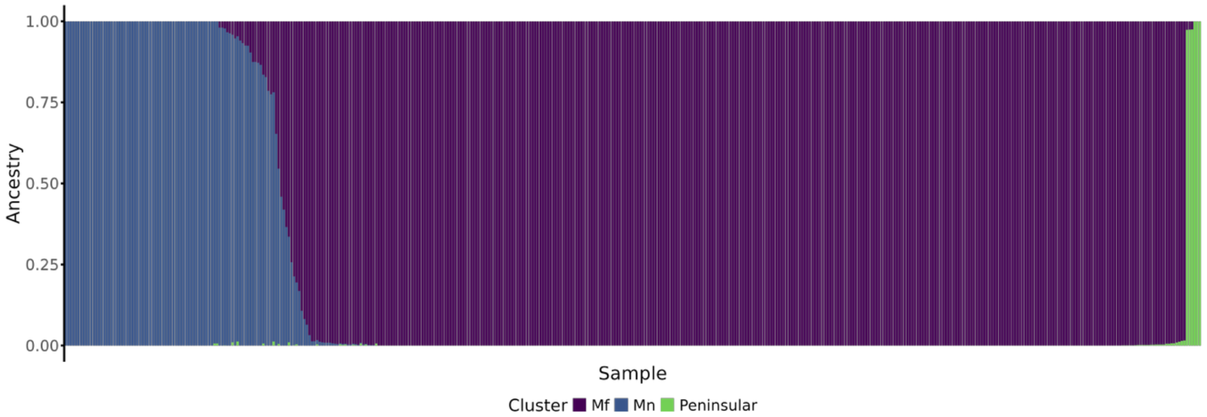

Supplementary Figure 1. Inferred population structure of *P. knowlesi* isolates based on ADMIXTURE clustering. Each vertical bar represents an individual isolate, with colours indicating proportional ancestry from inferred ancestral populations. Clustering highlights distinct genetic substructure consistent with geography and/or host origin.

Group	n	Male, n (%)	Female, n (%)	Age (years), median (IQR)	Parasitemia, median (IQR)
All	425	334 (78.6)	90 (21.2)	41.0 (26.0-51.0)	9.07e+03 (4.36e+03-2.64e+04)
Severe	112	92 (82.1)	20 (17.9)	47.5 (36.8-60.0)	4.74e+04 (1.65e+04-1.44e+05)
Uncomplicated	313	242 (77.3)	70 (22.4)	37.0 (24.0-49.0)	6.59e+03 (3.79e+03-1.38e+04)

Supplementary Table 1. Clinical and demographic characteristics of participants included in genetic analyses of *Plasmodium knowlesi* infection. Severe malaria was defined according to WHO criteria for *P. knowlesi* (Methods). Values are presented as n (%) or median (IQR).

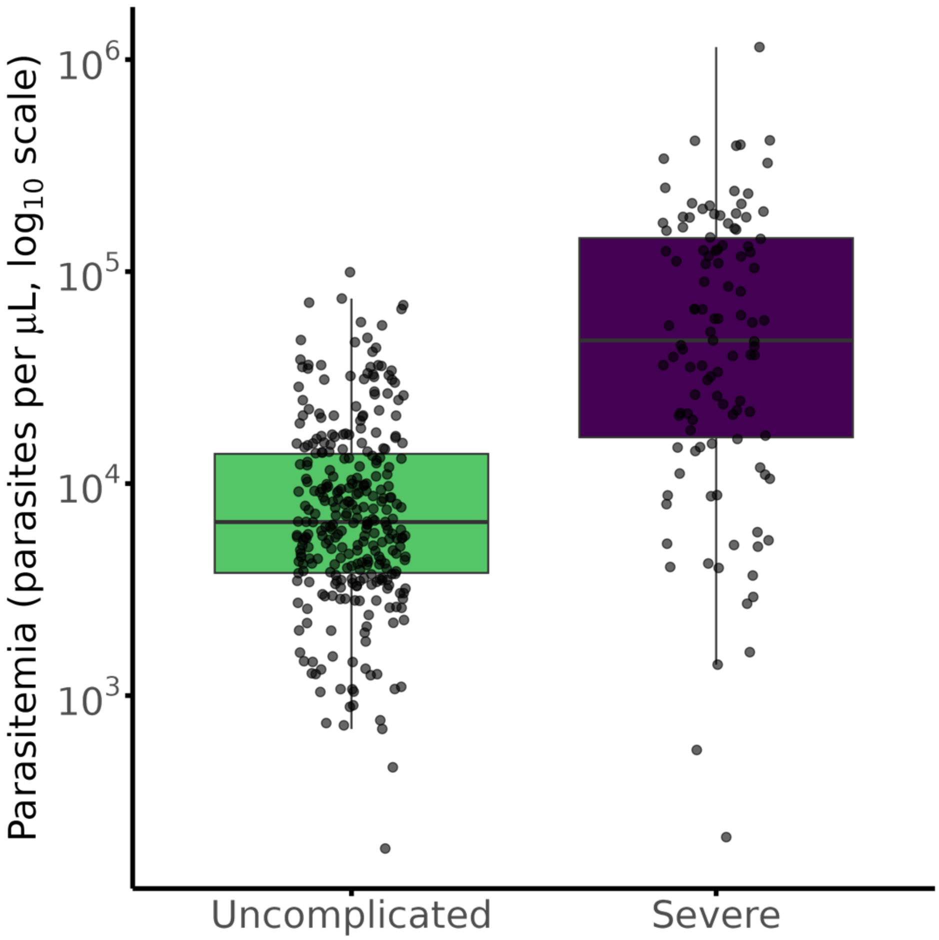

Supplementary Figure 2. Parasitemia differs between severe and uncomplicated Plasmodium knowlesi infections. Parasitemia (parasites per μL) is shown for patients with severe and uncomplicated malaria. Points represent individual patients and boxes indicate the median and interquartile range. Parasitemia was significantly higher in severe infections compared with uncomplicated infections (Wilcoxon rank-sum test, $P < 0.0001$), although substantial overlap between groups was observed.

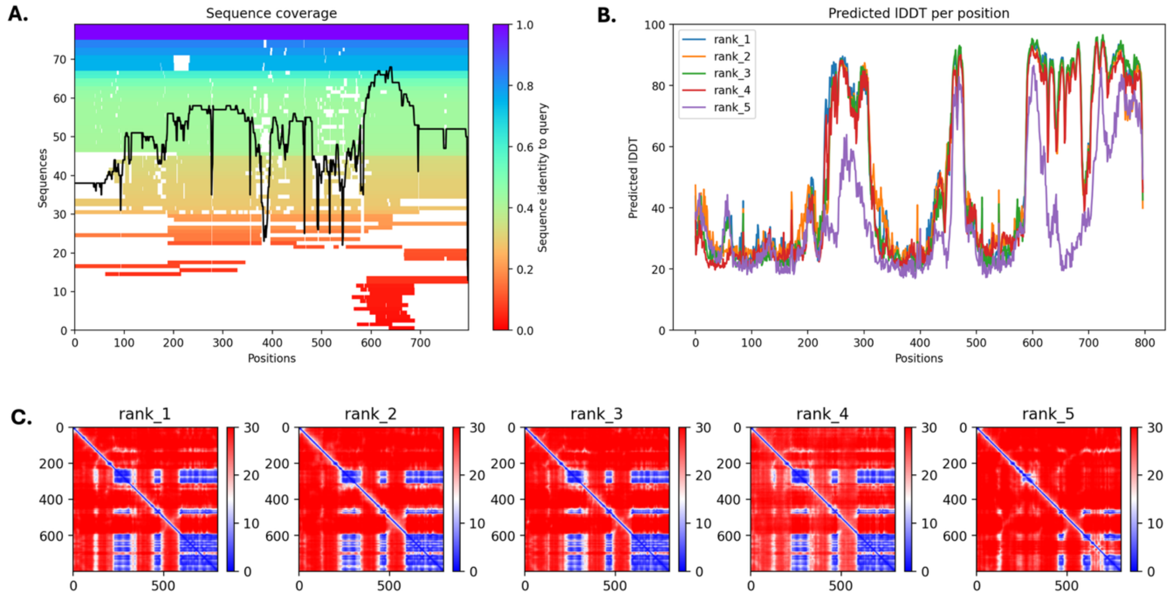

Supplementary Figure 3. Structural prediction and confidence metrics for uncharacterized P. knowlesi protein PKA1H_070012000. (**A**) Multiple sequence alignment (MSA) coverage and sequence identity for each aligned homolog used in structure prediction, with black line indicating number of aligned sequences per residue. (**B**) Per-residue predicted confidence scores (pLDDT) across five AlphaFold2 models, highlighting multiple high-confidence folded domains (pLDDT > 90). (**C**) Predicted Aligned Error (PAE) matrices for all five ranked models, indicating domain-level independence and potential modular structure.

Target	Scientific Name	Prob.	Seq. Id.	E-Value	Score	Query Pos.
A0A481Z9L5	Pithovirus LCPAC304	1	6.2	1.94E-06	210	591-764
A0A7D3UQY4	Fadolivirus algeromassiliense	1	12.5	1.84E-06	198	564-760
A0A2H4UVA7	Bodo saltans virus	1	11.2	7.30E-05	174	593-764
A0A3G4ZUJ5	Barrevirus sp.	1	9.6	6.22E-05	168	591-761
A0A3G5ADB4	Satyrvirus sp.	1	13.2	1.84E-06	159	572-762
M1IM71	Acanthocystis turfacea Chlorella virus OR0704.3	1	10.9	8.06E-04	123	593-762
A0A0P0YMZ6	Yellowstone lake phycodnavirus 2	0.99	11.3	2.11E-03	85	593-788
A0A8T9VR71	Iridoviridae	0.97	11	5.22E-03	75	599-791

Supplementary Table 2. High-confidence structural matches for PKA1H_070012000 identified by FoldSeek using AlphaFold2-predicted structures. Structural matches were retained if FoldSeek probability >0.9 and overlapping regions had AlphaFold pLDDT >90. For each protein, model confidence, aligned region, structural hit, database source, and putative functional annotation are reported.

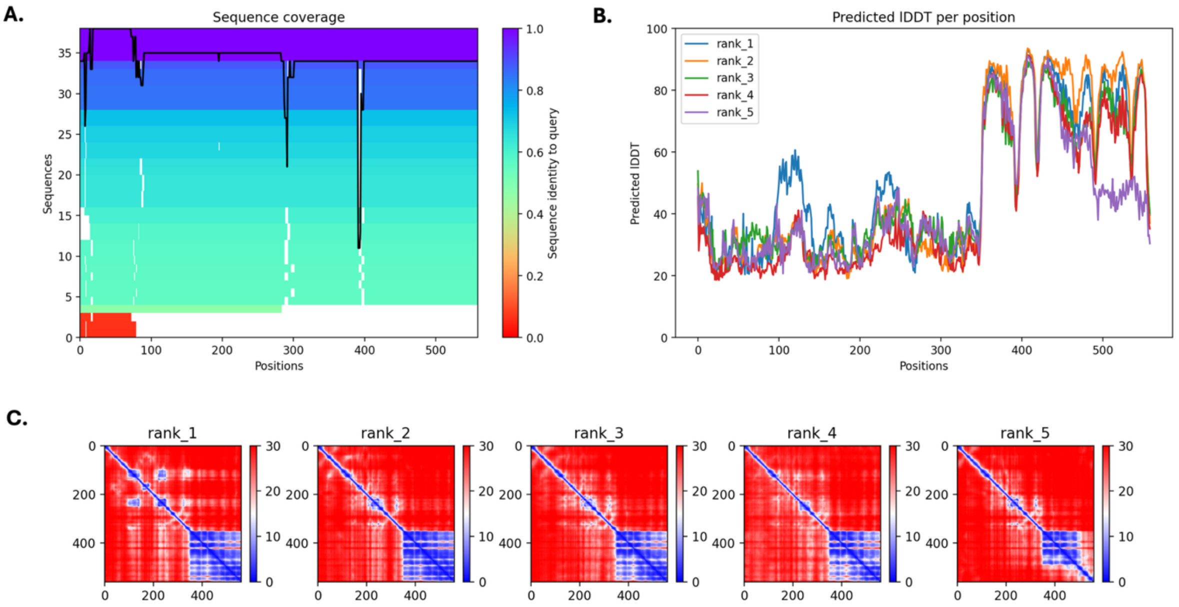

Supplementary Figure 4. Structural prediction and confidence metrics for uncharacterized P. knowlesi protein PKA1H_070016000. (**A**) Multiple sequence alignment (MSA) coverage and sequence identity for each aligned homolog used in structure prediction, with black line indicating number of aligned sequences per residue. (**B**) Per-residue predicted confidence scores (pLDDT) across five AlphaFold2 models, highlighting a single high-confidence folded domain (pLDDT > 90). (**C**) Predicted Aligned Error (PAE) matrices for all five ranked models, indicating domain-level independence and potential modular structure.

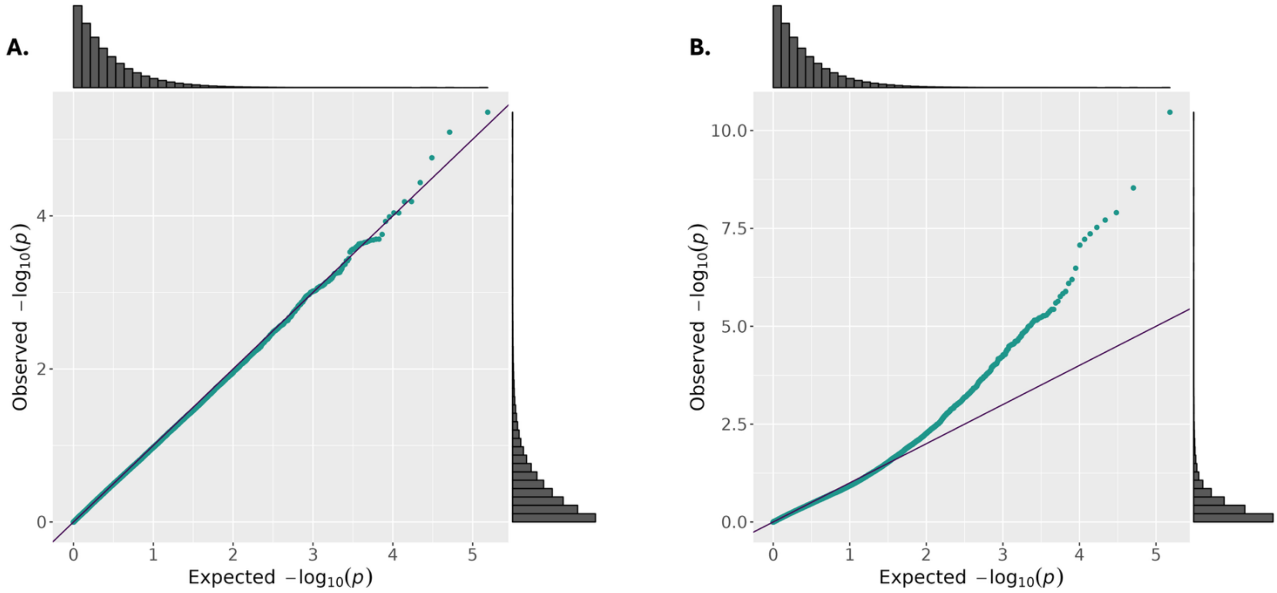

Supplementary Figure 5. QQ plots of transcriptome-wide differential expression analyses for severity and parasitemia. (A) Quantile–quantile (QQ) plot of observed versus expected p-values from the consensus differential expression model comparing severe versus uncomplicated malaria. The distribution largely follows the null, with deviation among the most significant transcripts consistent with biological signal ($\lambda = 0.998$). (B) QQ plot of p-values from models testing correlation between transcript expression and parasitemia. The upward deflection indicates widespread transcriptional association with parasite load, consistent with biological relevance of parasitemia-linked expression patterns ($\lambda = 0.960$). Marginal histograms show the distribution of observed $-\log_{10}(p)$ values in each analysis.

Metric	Reference genome	De novo transcriptome
Median input reads	7,209,880	7,209,880
Input reads (Q25)	4,778,271	4,778,271
Input reads (Q75)	20,729,931.75	20,729,931.75
Median mapped reads	1,957,322.5	4,031,267
Mapped reads (Q25)	352,252.5	1,623,161.25
Mapped reads (Q75)	8,844,753.5	16,024,962.25
Median mapped (%)	27.6	56.7
Mapped % (Q25)	8.7	34.1
Mapped % (Q75)	48.6	82.6

Supplementary Table 3. Comparison of read mapping rates to the P. knowlesi reference genome and de novo assembled transcriptome. Mapping statistics summarised across 210 clinical RNA-seq samples. Values represent the median and interquartile range (IQR; 25th–75th percentile) for input reads, mapped reads, and mapping percentages. Mapping to the de novo assembled transcriptome substantially increased read recovery relative to mapping to the reference genome.

Dataset	Median library size (Q1–Q3)	Median genes detected (Q1–Q3)
Raw	3,631,804 (1,379,695–16,137,502)	66,612 (49,361–97,722)
Filtered	543,392 (180,367–1,803,308)	7,250 (6,207–7,938)

Supplementary Table 4. Sequencing and transcript detection metrics before and after filtering. Median (Q1–Q3) per-sample values are shown for library size (total assigned reads),

percentage mapped reads (reference and de novo assemblies), and number of detected transcripts (≥1 read), comparing the raw dataset and the final filtered analysis dataset.

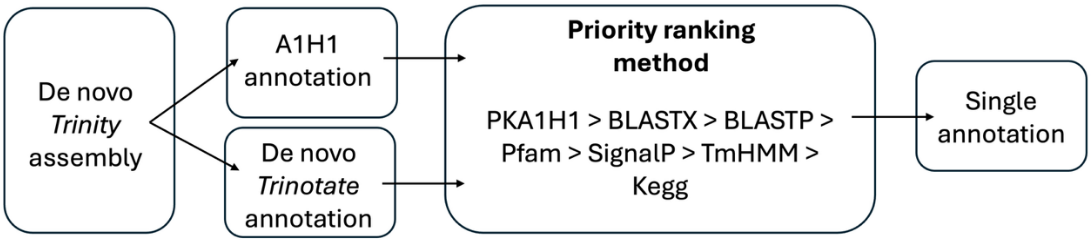

Supplementary Figure 6. Annotation workflow showing integration of de novo *Trinity* assembly with PKA1H1 reference-guided and *Trinotate* annotation, prioritised by a structured ranking method (A1H1 > BLASTX > BLASTP > Pfam > SignalP > TmHMM > KEGG).

Life stage	W (on)	p-value (Wilcoxon)	Rho (Spearman)	p-value (Spearman)
Ring	2951	0.26	-0.12	0.08
Schizont	3121	0.53	-0.03	0.64
Trophozoite	3433	0.77	0.06	0.35
Gametocyte, developing	3691	0.30	-0.01	0.84
Gametocyte, female	3814	0.16	0.13	0.07
Gametocyte, male	3448	0.74	-0.02	0.76

Supplementary Table 5. Results for statistical testing of associations between Scaden-inferred IDC life stages and both severity and parasitemia. *P. knowlesi* reference data was used for the asexual prediction and *P. berghei* for the sexual stages (1). Associations with severity were tested using Wilcoxon rank-sum tests and Spearman correlation analysis for $\log_{10}$-transformed parasitemia.

Life stage	W (Wilcoxon)	p-value (Wilcoxon)	Rho (Spearman)	p-value (Spearman)
Ring	4175	**0.01**	-0.02	0.80
Schizont	3535	0.56	0.05	0.45
Trophozoite	2639	0.04	0.01	0.92

Gametocyte, developing	2696	0.06	-0.01	0.91
Gametocyte, female	3240	0.78	0.14	**0.05**
Gametocyte, male	2894	0.20	-0.01	0.90

Supplementary Table 6. Results for statistical testing of associations between Scaden-inferred IDC life stages and both age and sex. *P. knowlesi* reference data was used for the asexual prediction and *P. berghei* for the sexual stages (1). Associations with sex were tested using Wilcoxon rank-sum tests and Spearman correlation analysis for log_{10}-transformed age.

Parasitemia	Gametocyte, developing	Gametocyte, female	Gametocyte, male
0	0.05	0.08	0.06
52	0.14	0.14	0.21
291	0.20	0.11	0.14
1040	0.03	0.04	0.03
1044	0.06	0.07	0.07
1175	0.09	0.08	0.13
1262	0.01	0.02	0.02
1274	0.03	0.05	0.03
1400	0.06	0.08	0.06
1440	0.03	0.05	0.04

Supplementary Table 7. Low parasitemia (< 5000 parasites/µL) samples with proportions of gametocytes predicted using Scaden with the *P. berghei* reference data. Parasitemia is reported as parasites per microliter.

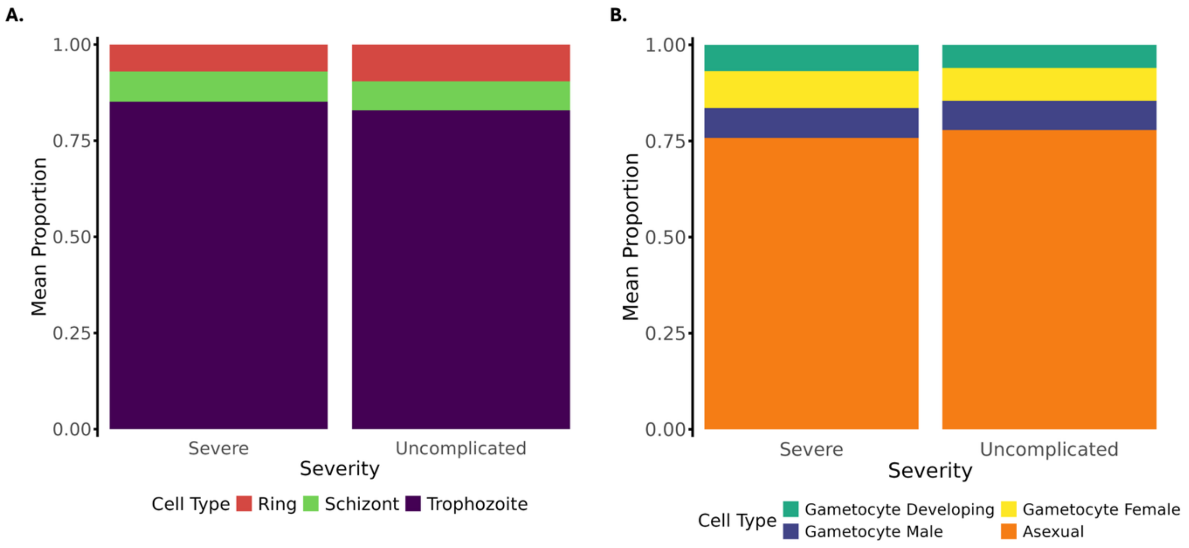

Supplementary Figure 7. Mean Intraerythrocytic developmental cycle (IDC) composition of *Plasmodium knowlesi* infections. Proportions of mean stage composition stratified by severe and uncomplicated infections, showing predicted gametocyte life stage proportions estimated with Scaden using *P. knowlesi* **(A)** and *P. berghei* **(B)** single cell reference sets.

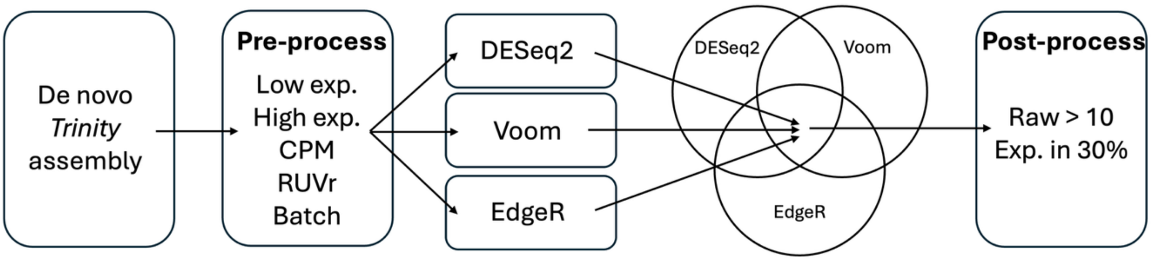

Supplementary Figure 8. Differential expression (DE) analysis pipeline combining DESeq2, edgeR, and voom, with consensus outputs.

PC	Variable	R^2 (linear model)	p-value (linear model)	Rho (Spearman)	p-value (Spearman)
1	Library size	0.011	0.127	-0.097	0.161
2	Library size	0.013	0.093	-0.166	0.016
3	Library size	0.002	0.531	0.027	0.7
1	Transcripts detected	0.092	< 0.001	0.292	< 0.001

2	Transcripts detected	0.05	0.001	-0.231	0.001
3	Transcripts detected	0.49	< 0.001	0.575	< 0.001
1	Mapped reads	0.027	0.017	0.159	0.022
2	Mapped reads	0.054	0.001	-0.228	0.001
3	Mapped reads	0.137	< 0.001	0.181	0.009

Supplementary Table 8. Correlation analyses comparing relationship between technical factors and principal components. Results for several linear models and Spearman correlation analysis for library size, transcript counts and mapped reads vs the first three principal components.

PC	Variable	R^2 (linear model)	p-value (linear model)	Rho (Spearman)	p-value (Spearman)
1	Schizont	0.015	0.08	-0.105	0.13
2	Schizont	0	0.976	-0.026	0.704
3	Schizont	0.047	0.002	-0.001	0.984
1	Ring	0.083	< 0.001	-0.437	< 0.001
2	Ring	0.05	0.001	-0.124	0.073
3	Ring	0	0.934	0.025	0.718
1	Trophozoite	0.089	< 0.001	0.257	< 0.001
2	Trophozoite	0.036	0.006	-0.084	0.228
3	Trophozoite	0.01	0.153	-0.055	0.426
1	Developing gametocyte	0.063	< 0.001	0.318	< 0.001
2	Developing gametocyte	0.063	< 0.001	0.271	< 0.001
3	Developing gametocyte	0.058	< 0.001	-0.084	0.225
1	Female gametocyte	0.038	0.005	0.251	< 0.001
2	Female gametocyte	0.001	0.639	0.139	0.044
3	Female gametocyte	0.017	0.062	-0.064	0.355
1	Male gametocyte	0.042	0.003	0.328	< 0.001
2	Male gametocyte	0.026	0.02	0.256	< 0.001
3	Male gametocyte	0.121	< 0.001	-0.186	0.007

Supplementary Table 9. Correlation analyses comparing relationship between life stage proportions and principal components. Summary of potential associations for PC1-3 differentially between different life stage proportions inferred with scaden and using *Malaria Cell Atlas* single cell reference sets for *P. knowlesi* (rings, schizonts and trophozoites) and *P. berghei* (gametocytes).

Upregulated	Protein	Adjusted *p*	LogFC	LogFC SE
Severe	unspecified product	0.033	2.879	0.612
	high mobility group protein B2, putative	0.039	1.398	0.338
	CCR4-NOT transcription complex subunit 1, putative	0.039	1.340	0.325
	hypothetical protein, conserved	0.039	1.432	0.355
	unspecified product	0.039	2.868	0.711
	meiotic recombination protein DMC1, putative	0.039	1.419	0.356
	blood-stage antigen 41-3, putative	0.039	1.340	0.333
	calpain, putative	0.039	1.814	0.453
	AAA family ATPase, putative	0.039	1.095	0.276
	erythrocyte membrane-associated antigen, putative	0.04	1.293	0.328
	28 kDa ookinete surface protein, putative	0.041	1.769	0.451
	formin 2, putative	0.044	1.139	0.296
	subtilisin-like protease 2, putative	0.044	1.113	0.289
	LCCL domain-containing protein	0.046	1.377	0.366
	reductase, putative	0.046	1.235	0.332
Uncomplicated	Protein-L-isoaspartate O-methyltransferase domain-containing protein 1	0.033	-2.021	0.435
	Cyclin-dependent kinase 19	0.035	-1.518	0.348
	Histone-lysine N-methyltransferase 2C	0.035	-1.421	0.328
	Protein phosphatase 1B	0.035	-1.544	0.354
	MORC family CW-type zinc finger protein 3	0.038	-1.973	0.464
	SAFB-like transcription modulator	0.038	-1.917	0.452
	SWI/SNF-related matrix-associated actin-dependent regulator of chromatin subfamily A member 5	0.039	-1.586	0.398
	Zinc finger CCCH domain-containing protein 6	0.039	-1.681	0.410
	Serine/threonine-protein kinase tousled-like 1	0.039	-1.427	0.353
	RNA-binding protein 33	0.039	-1.874	0.447
	Zinc finger protein 236	0.039	-1.608	0.395

	Regulator of cell cycle RGCC	0.039	-1.878	0.466
	Transcription factor Dp-2	0.039	-1.420	0.349
	Cyclin-dependent kinase 17	0.039	-1.573	0.391
	MAP/microtubule affinity-regulating kinase 3	0.039	-1.594	0.402

Supplementary Table 10. Top 15 differentially expressed parasite genes for uncomplicated and severe *P. knowlesi* malaria with brief, tentative biological description. Genes with significant expression differences (FDR-adjusted $p < 0.05$) are shown, with annotation source indicated. Full results including log2 fold changes, standard errors, all annotations, mutability metrics, and more, for all 130 significant transcripts (112 annotated) are provided in Supplementary File 3.

Gene	Description	p-adj (voom)	logFC (voom)	Source
TRINITY_DN680050_c0_g1	NA	<0.001	0.792	NA
TRINITY_DN112057_c8_g1	NA	<0.001	0.784	NA
TRINITY_DN949463_c0_g1	NA	<0.001	0.723	NA
TRINITY_DN762379_c0_g1	NA	<0.001	0.844	NA
TRINITY_DN47323_c0_g1	28 kDa ookinete surface protein, putative	<0.001	0.594	PKA1H1
TRINITY_DN9599_c0_g1	reductase, putative	<0.001	0.536	PKA1H1
TRINITY_DN1381511_c4_g1	NA	<0.001	0.974	NA
TRINITY_DN299291_c1_g1	unspecified product	<0.001	0.821	PKA1H1
TRINITY_DN11906_c0_g2	NA	<0.001	0.520	NA
TRINITY_DN14557_c0_g1	erythrocyte membrane-associated antigen, putative	<0.001	0.575	PKA1H1
TRINITY_DN38550_c1_g1	NA	<0.001	0.740	NA
TRINITY_DN279326_c2_g1	unspecified product	<0.001	0.702	PKA1H1
TRINITY_DN35871_c0_g1	meiotic recombination protein DMC1, putative	<0.001	0.519	PKA1H1
TRINITY_DN360328_c22_g1	NA	<0.001	0.620	NA
TRINITY_DN25702_c0_g1	hypothetical protein, conserved	<0.001	0.526	PKA1H1
TRINITY_DN14177_c0_g1	AAA family ATPase, putative	<0.001	0.534	PKA1H1
TRINITY_DN99357_c0_g1	calpain, putative	<0.001	0.629	PKA1H1
TRINITY_DN31815_c0_g1	conserved Plasmodium protein, unknown function	<0.001	0.474	PKA1H1
TRINITY_DN11069_c0_g1	erythrocyte membrane-associated antigen, putative	<0.001	0.487	PKA1H1

TRINITY_DN11835_c0_g1	conserved Plasmodium protein, unknown function	<0.001	0.543	PKA1H1
TRINITY_DN10352_c0_g1	conserved Plasmodium protein, unknown function	<0.001	0.471	PKA1H1
TRINITY_DN1116_c0_g1	conserved Plasmodium protein, unknown function	<0.001	0.476	PKA1H1
TRINITY_DN1350_c0_g1	conserved Plasmodium protein, unknown function	<0.001	0.514	PKA1H1
TRINITY_DN4936_c0_g1	blood-stage antigen 41-3, putative	<0.001	0.492	PKA1H1
TRINITY_DN4045_c2_g1	NA	<0.001	0.492	NA
TRINITY_DN16271_c0_g1	high mobility group protein B2, putative	<0.001	0.476	PKA1H1
TRINITY_DN50536_c0_g1	subtilisin-like protease 2, putative	<0.001	0.506	PKA1H1
TRINITY_DN10699_c1_g1	conserved Plasmodium protein, unknown function	<0.001	0.547	PKA1H1
TRINITY_DN18062_c0_g1	conserved Plasmodium protein, unknown function	<0.001	0.547	PKA1H1
TRINITY_DN19359_c0_g1	LCCL domain-containing protein	<0.001	0.494	PKA1H1

Supplementary Table 11. Overlapping differentially expressed genes for severity and parasitemia models. Parasitemia model was performed as described in the methods, except with the continuous log_{10}-transformed parasitemia term in place of severity. The limma/voom statistics are presented here, along with the top annotation (prioritisation methods described in methods).

gene	IDC life stage	Rho
TRINITY_DN299291_c1_g1	Gametocyte developing	0.59
TRINITY_DN949463_c0_g1	Ring	-0.54
TRINITY_DN949463_c0_g1	Gametocyte, total	0.53
TRINITY_DN949463_c0_g1	Gametocyte, male	0.53
TRINITY_DN299291_c1_g1	Gametocyte, male	0.58
TRINITY_DN949463_c0_g1	Gametocyte, developing	0.52
TRINITY_DN299291_c1_g1	Gametocyte, total	0.57
TRINITY_DN360328_c22_g1	Gametocyte, male	0.56
TRINITY_DN360328_c22_g1	Gametocyte, total	0.53
TRINITY_DN299291_c1_g1	Ring	-0.52
TRINITY_DN360328_c22_g1	Gametocyte, developing	0.51

Supplementary Table 12. Life cycle stage associations of differentially expressed transcripts. Summary of 11 transcripts significantly differentially expressed between severe and uncomplicated *P. knowlesi* infections that are putatively associated with specific life cycle stages predicted with Scaden and *P. knowlesi* (asexual stages) and *P. berghei* (sexual stages) reference data. All associations passed multiple-testing correction (adjusted $p < 0.0001$).

Wilcoxon rank-sum test	
W	28426
P-value	< 0.001

Supplementary Table 13. Wilcoxon rank-sum test comparing parasitemia between severe and uncomplicated malaria cases. Parasitemia values were $\log_{10}$-transformed prior to testing.

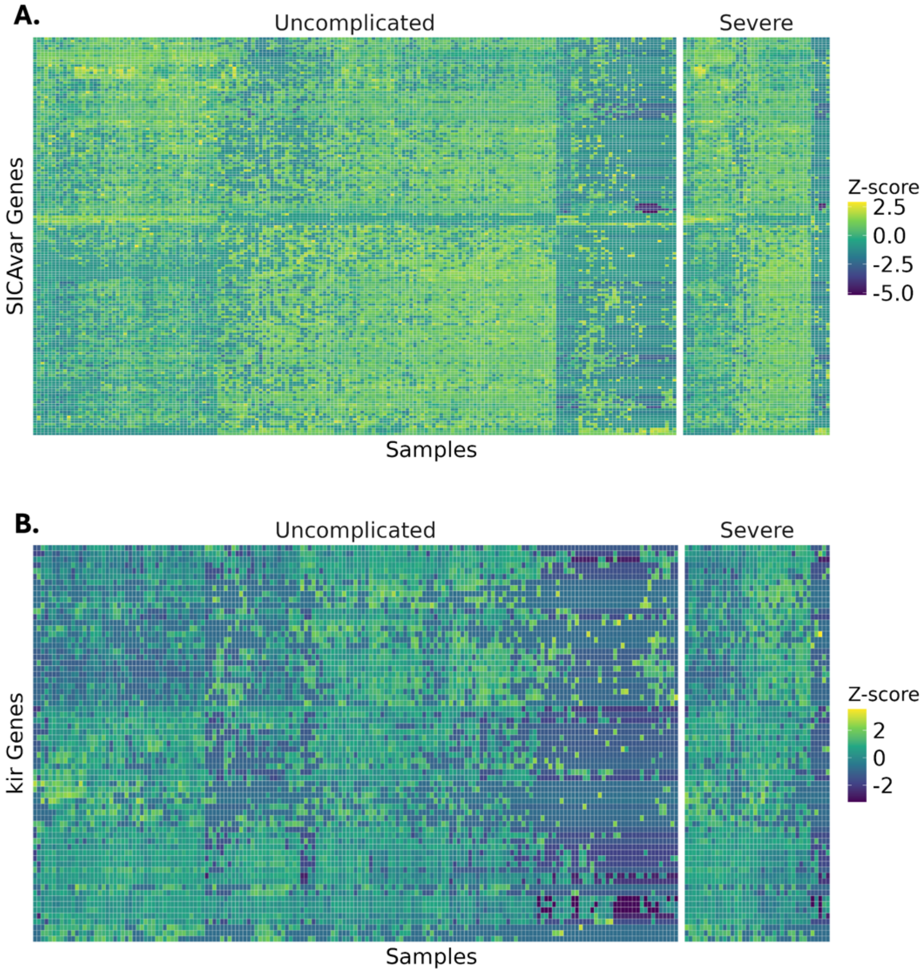

Supplementary Figure 9. Targeted heatmaps of SICAvar and KIR gene families reveals heterogeneity across families, despite elevated expression in severe malaria. (A) Sample-

level z-score heatmap of filtered SICAvar gene expression across all samples. Columns represent individual patients, and rows represent filtered SICAvar transcripts (logCPM >10 in ≥30% of samples). Z-scores were calculated per transcript across all samples. Both gene-level and grouped-level heatmaps were clustered to highlight expression patterns. (B) Sample-level z-score heatmap of KIR gene expression using the same filtering and normalization approach as panel A. Z-scores were calculated using logCPM-normalized expression values after gene-wise centering and scaling.

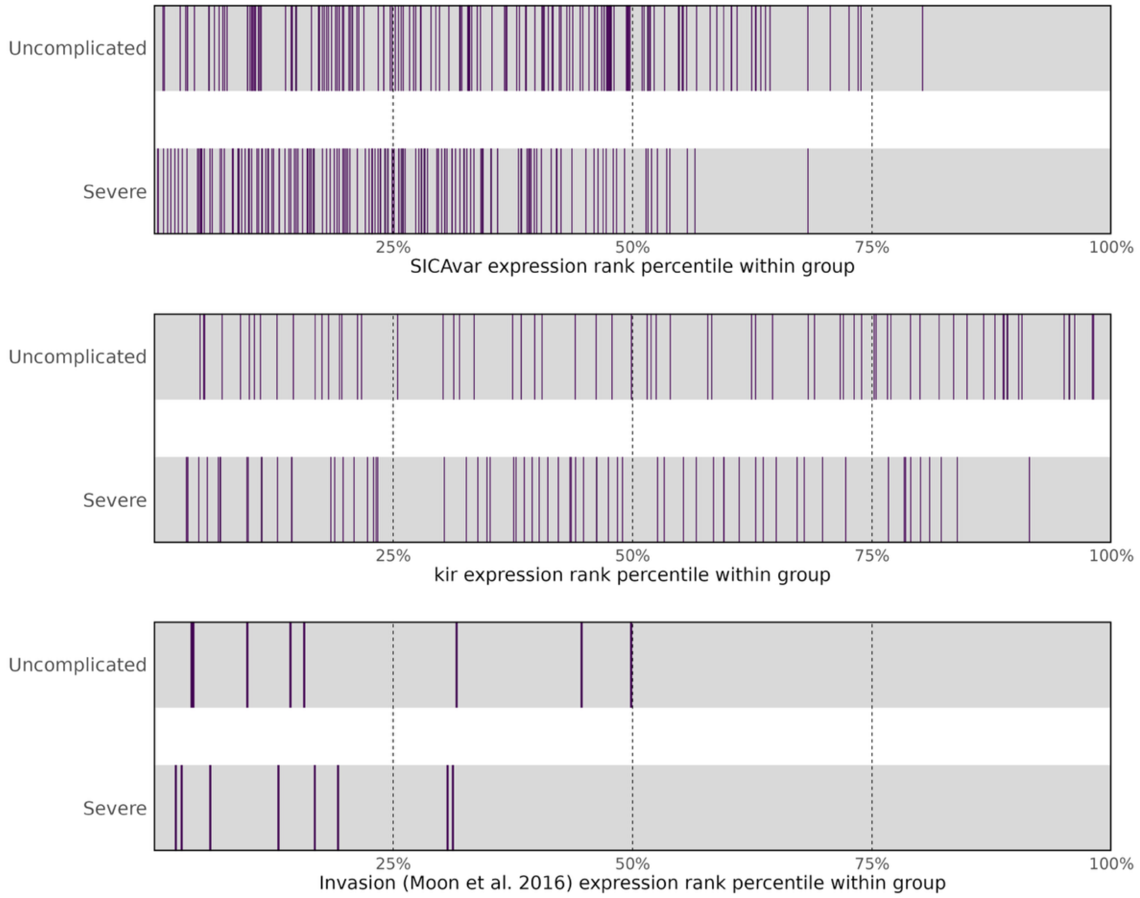

Supplementary Figure 10. Global expression rank distribution of targeted gene families across clinical severity groups. Barcode plots show the relative expression rank percentiles of transcripts or genes of interest within the full parasite transcriptome, computed separately for uncomplicated and severe infections. For each group, all expressed transcripts were ranked by mean logCPM expression, and the positions of target features are shown as vertical bars. Top: SICAvar transcripts; middle: kir transcripts; bottom: invasion-related genes curated from Moon et al. (2016), collapsed to gene-level expression prior to ranking. Grey bands represent

the full ranked transcriptome, with dashed lines indicating the 25th, 50th, and 75th expression percentiles.

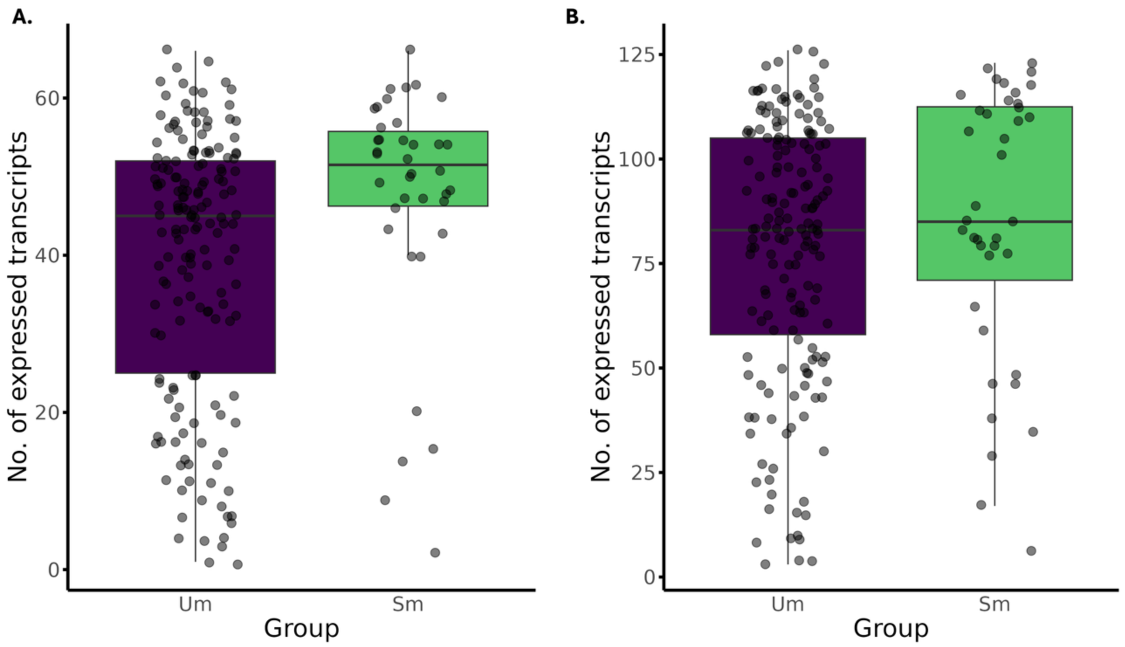

Supplementary Figure 11. Per-sample kir and SICAvar transcriptional burden in severe and uncomplicated malaria. (A) Number of kir transcripts expressed per infection in uncomplicated malaria (Um) and severe malaria (Sm) cases. **(B)** Number of SICAvar transcripts expressed per infection in uncomplicated and severe malaria cases. Each point represents an individual infection; boxplots indicate the median and interquartile range, with whiskers extending to 1.5× the IQR. Both gene families show higher transcriptional burden in severe malaria, with kir exhibiting a significantly greater number of expressed transcripts per infection compared with uncomplicated cases (Wilcoxon test; see Results).

Analysis	Test Type	Result	Adjusted
Fever vs Severe Malaria	Wilcoxon rank-sum test	p = 0.2311, W = 2907.5	No
Temperature vs Parasitemia	Spearman correlation	p = 0.16, rho = 0.0975405	No

Temperature vs Expression of 'Stress' Genes (logCPM expression of curated heat-related transcripts [GO terms])	Spearman correlation per gene	No significant hits.	Yes

Supplementary Table 14. Summary of targeted statistical analyses examining the relationship between fever, parasitemia, and heat-responsive gene expression in Plasmodium knowlesi infections. Gene sets were derived from GO annotations linked to heat stress; both 'heat' search in GO terms and curated list from a publication by Zhang et. al. (2). Significance was assessed using non-parametric tests and adjusted for multiple testing where applicable.

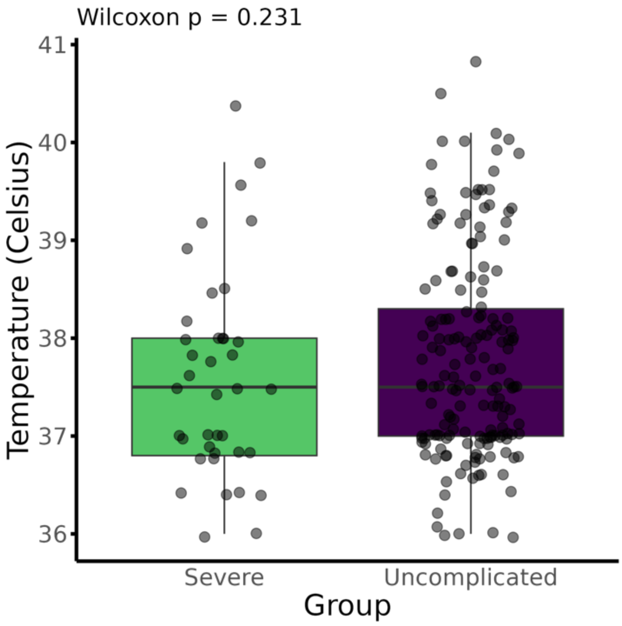

Supplementary Figure 12. Boxplot comparing the distributions of temperature at admission of patients with severe and uncomplicated malaria.

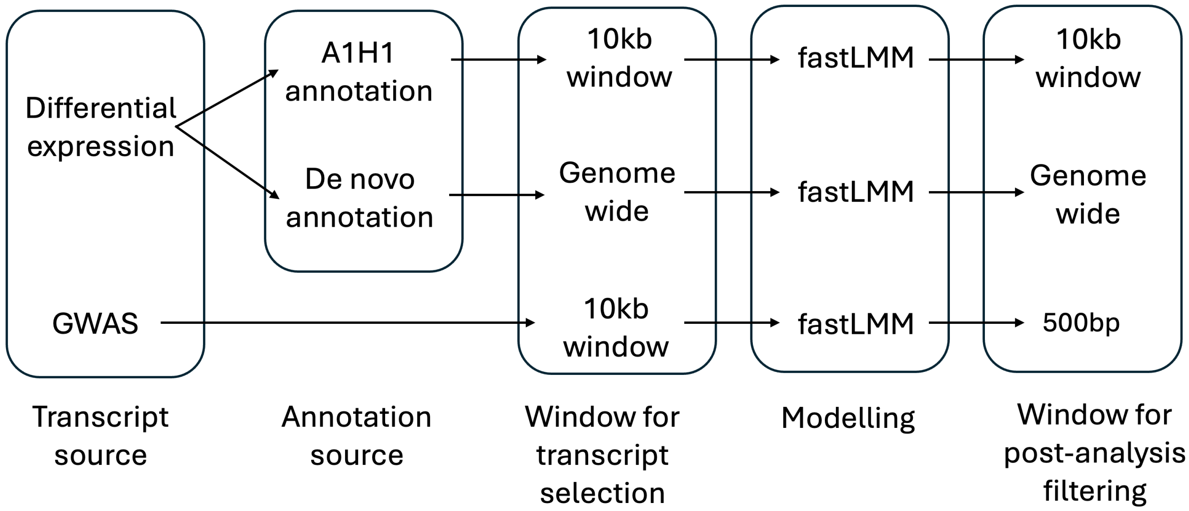

Supplementary Figure 13. Overview of the integrative framework used to identify expression quantitative trait loci (eQTLs) in Plasmodium knowlesi. Schematic of the analytical workflow integrating GWAS, differential expression, transcriptome annotation, and multiple statistical modelling approaches to prioritise cis- and trans-eQTL signals. All statistical thresholds and covariates are described in Methods.

1. Howick VM, Russell AJC, Andrews T, Heaton H, Reid AJ, Natarajan K, et al. The Malaria Cell Atlas: Single parasite transcriptomes across the complete Plasmodium life cycle. Science. 2019;365(6455):eaaw2619.

2. Zhang M, Wang C, Oberstaller J, Thomas P, Otto TD, Casandra D, et al. The apicoplast link to fever-survival and artemisinin-resistance in the malaria parasite. Nature Communications. 2021;12(1):4563.
